# Antitumor activity of withaferin-A and propolis in benz (a) pyrene-induced breast cancer

**DOI:** 10.6026/97320630018841

**Published:** 2022-09-30

**Authors:** Thazhathuputhenpurayil Sadhasivan Meghalatha, Natarajan Muninathan

**Affiliations:** 1 Central Research Laboratory, Meenakshi Medical College Hospital and Research Institute, Meenakshi Academy of Higher Education and Research, Kanchipuram Tamil Nadu, India – 631552

**Keywords:** Breast cancer, withaferin-A, propolis, Benz(a)pyrene, carcino embryonic antigen (CEA)

## Abstract

Female breast cancer is the leading malignancy surpassing lung cancer recently, and its incidence is continued to rise in many countries. The existing anticancer drugs have limitations like drug resistance and adverse effects leading to poor clinical outcomes. The natural compounds withaferin-A and propolis have been individually reported for their anticancer activity in preclinical models. However, the combined effect of these compounds has not been studied especially in breast cancer models. Therefore, it is of interest to evaluate the effect of Withaferin-A and propolis on Benz(a)pyrene-induced breast cancer. Wistar rats of female gender were treated with saline (normal control), Benz(a)pyrene (disease control), Benz(a)pyrene+ Withaferin-A or Propolis, Benz(a)pyrene+ Withaferin-A+ Propolis. At the end of the treatment, the plasma levels of carcino embryonic antigen (CEA) were measured. We observed a decrease in carcino embryonic antigen (CEA) levels in rats received withaferin-A and propolis combination rather than individual compounds indicating their beneficial role in breast cancer. Results of the present study show that propolis, when combined with withaferin A, exhibits better anti tumor activity than its individual effect in Benz (a) pyrene-induced mammary carcinogenesis.

## Background:

Cancer is a prime health hassle with an increasing trend of occurrence worldwide. Nearly two crore new cases and one crore cancer-related death were reported in 2020 across the globe. Among all cancers, female breast cancer reported a high incidence with 23 lakh newly diagnosed cases and became the highest reported malignancy. Around 12% of the Indian population is at risk of developing cancer. Mortality rates of female breast cancer are high in transitioning countries. Risk factors like family history, genetic factors, age, geographical location, associated health issues, and nutritional and lifestyle modifications predisposed breast cancer incidence [1]. Breast cancer is the most frequent in metropolitan cities, and this could be because of exposure to many cancer-causing agents. Lack of awareness and late diagnosis is responsible for most female reproductive system-related cancers, especially breast cancer [2]. However, public and private organizations are bringing awareness to undercut the disease incidence. On the other hand, currently available anticancer drugs fail to offer a complete cure for female breast cancer besides treatment-related toxicities [3]. This limitation with conventional anticancer drugs paves the way to screen effective and save natural compounds against breast malignancy. A compound from the Indian traditional plant, Ashwagandha, scientifically called Withania somnifera L. Dunal has had many indications in Ayurvedic medicine for ages. Withania somnifera exhibits multiple pharmacological effects in experimental animals. Its active constitute, withaferin A (WA) shown to produce anticancer effects in experimental cancer models. The expression of NF-kB regulatory genes in cancer cells has been inhibited by WA [4]. Breast cancer cells show hyperactive signaling pathways which further propagate tumor growth. Modulation of many signaling pathways, like interleukin-6, transcription 3 is responsible for anticancer activity of WA. This compound acts as an apoptosis inducer and exhibits anti-migratory activities in breast cancer cells [5]. Also, WA was reported to decrease the proliferating cell nuclear antigen (PCNA) expression, thereby suppressing the proliferation of human breast cells [6].

A complex natural matrix that honey bees collect from tree buds, saps and other plant sources and is mixed with its saliva is called Propolis [7]. Caffeic acid phenethyl ester-CAPE, an active ingredient isolated from ethanolic extract of propolis has been reported for many pharmacological properties. Its ability to induce cytotoxicity, apoptosis, autophagy and prevent inflammation by modulating TLR-4 signalling pathway has collectively contributed to anticancer properties in breast cancer cells [8-10]. Moreover, it has been demonstrated that breast cancer cells treated with propolis and its active ingredient (cardanol) modulate cell signaling pathways like PI3K/Akt, p38 MAPK and ERK1/2 and induce apoptosis [11]. Propolis has been reported for its anti proliferative effect by inducing G0/G1 and G2/M phase of cell cycle arrest. Also, the upregulated expression of p21 and p27 was observed with propolis treatment in experimental cancer models [12] Propolis can also suppress the side effects caused by chemotherapy with a curative effect [13]. By enhancing the radio sensitivity of breast cancer cells, Propolis potently interfere with Ribo nucleotide reductase (RNR) function [14]. Serum carcino embryonic antigen (CEA) is the widely studied tumor marker in breast cancer [15]. CEA had a more important prognostic value for primary breast cancer before treatment. Elevated CEA levels are closely related to age and tumor burden [16]. Therefore, it is of interest to evaluate the CEA levels to determine the antitumor activity of Withaferin-A & Propolis on Benz(a)Pyrene-induced breast cancer in Wistar rats.

## Materials & Methods:

### Ethical approval:

Institutional Animal Ethical Clearance was obtained on 03/07/2019 (IAEC No: 003/2019)

### Animals:

National Institute of Nutrition, Hyderabad, India, provided female Wistar rats (150-200gms) for the study. Animals were initially acclimatized to the new conditions before the initiation of experiments. They were maintained in controlled environmental conditions with optimal temperature and humidity on alternative 12 hr light/dark cycles. All the animals were fed a standard pelleted diet (Gold Mohr rat feed, MS. Hindustan lever Ltd. Mumbai) and water ad libitum. Withaferin-A, Propolis and Benz (a) Pyrene were procured from Sigma Aldrich, Mumbai, India and all other chemicals used in the study were purchased from SRL, Chennai, India.

### Experimental protocol:

Six rats were in each group, and a total of five groups were divided after screening the animals for any underlying pathology.

### Group I:

Non-disease group-normal control-received saline.

### Group II:

Disease control group-received benz(a)pyrene (20 mg in sunflower oil and saline, 0.5 ml each) in two mammary pads by "air pouch technique") weekly twice for 3months to induce breast cancer.

### Group III:

Intervention group-breast cancer-bearing animals treated with withaferin A orally (30mg/kg b.wt, ) weekly for four weeks.

### Group IV:

Intervention group-breast cancer-bearing animals treated with ethanolic extract of propolis orally (50 mg/Kg body weight) for 30 days.

### Group V:

Intervention group-Breast cancer-bearing animals treated with a combination of withaferinA and ethanolic extract of propolis (as above).

### Preparation of ethanolic extract of propolis:

Ethanolic extract of propolis (95% v/v) was prepared in a hermetically closed glass vessel placed at 37°C with occasional shaking for 4 days. The contents were filtered using Whatman filter paper (size 4), and the solvent was separated using a rotary evaporator set at 60°C.

### Induction of cancer:

#### Production of Air pouch:

We adopted a method suggested by previous researchers to make air pouches in experimental animals (Arun et al. 1984). Using a 5 ml syringe, about 2 ml of air was drawn into the syringe, and it was autoclaved. The air pouch was introduced just beneath the mammary fat pad subcutaneously by injecting the sterile air. The carcinogen was administered a day after inducing the air pouch for stabilization.

#### Administration of carcinogen:

Benz(a)pyrene20 mg in sunflower oil and saline, 0.5 ml each, were added to a vial and vortexed vigorously to obtain a uniformly dispersed emulsion. At least a day before, a single dose of Benz(a)pyrene was injected into the air pouch. Breast tumor growth was monitored regularly, and the maximum tumor size was attained within 90 days of carcinogen administration.

### Sample collection:

All experimental groups, including normal control animals, were sacrificed by cervical decapitation at the end of the study. Blood was collected in EDTA and plain tubes to collect both plasma and serum, respectively, for blood parameters. The ice-cold 0.1M Tris-HCl buffer (pH 7.4) was used for homogenizing liver and breast tissues with the help of a motor-driven teflon coated homogenizer.

## Results:

### CEA levels in the control group (Group-1) vs Group-II:

Compared to normal control (Group-I), breast cancer-bearing animals (Group-II) showed an elevation of CEA levels, indicating the development of breast cancer tumor in the disease control group.

### CEA levels in comparison to Group II and III:

While comparing the CEA levels of breast cancer-bearing animals (Group-II) with the animals of withaferin-A treated animals (Group-III) are having less levels of CEA(p<0.001), which indicates that the Withaferin-A shows its antitumor activity due to which the animals show prognosis in treatment.

### CEA levels in comparison to Group II & IV:

In comparison with the breast cancer-bearing animals (Group-II), CEA levels are in a reduced concentration in Propolis-treated animals (Group-IV) with a p<0. 001. This indicates that the Propolis shows its curative effect by its anti proliferative effect or increased apoptosis effect.

### CEA levels in comparison to Group II & V:

The CEA levels were markedly decreased in Withaferin A, and Propolis treated animals (Group-V) compared to breast cancer-bearing animals (Group-II). The treatment with a combination of Withaferin-A and Propolis effectively reduced CEA levels. It's a good sign of prognostic improvement with combination treatment.

## Discussion:

Decreased tissue CEA levels (p<0.001) were found in WA & Propolis treated animals compared to the untreated breast cancer-bearing animal group. This mechanism may be due to the anti-inflammatory and anticancer effects of both propolis & WA. The treatment with both in combination has more effects than without combination. Cancer research faces many challenges in reducing the side effects of chemotherapy to get maximum effect & non-toxic compounds that will protect the normal tissues from the chemotherapy. All these compounds should be protective in actions against mutation and genetic damage and resist the changes to the immune system. Apoptosis-related gene expression was found to decrease in the breast cancer study [11]. Propolis of seven stingless bees has shown cytotoxicity activity on cancer cell lines [17]. It has been reported that propolis may enhance the radiosensitivity of breast cancer cells by interfering with RNR function [14]. Another research group suggested that propolis is an effective food supplement and should be used by people in cancer treatment to improve their quality of life [18]. Propolis is natural compounds from bee wax with bioactive enzymes, terpenes, antioxidant flavonoids, phenolic acid, and phenolic compounds. These compounds promote health and have known for their beneficial role in many disorders [19]. Propolis has an anticancer effect by inhibiting the pathways for cancer initiation, progression and metastasis and also on intrinsic and extrinsic apoptosis pathways [20]. WA is a herbal which promises the inhibition of apoptosis in human breast cancer cells by mediating mitochondria as shown by Hahm (2011) [4] and can inhibit the cell migration even after the activation of STAT3 by Interleukin-6(IL-6), which is a beneficial effect of WA as shown by Lee (2010) [5]. A study was done by Behl et al. (2020) [21] says the beneficial treatment effect of WA on chemotherapic drugs & radiotherapy in combination with WA analogues by regulating the multiple antitumor pathways, inhibiting the cell proliferation, reducing angiogenesis progression and metastasis progression. Reduced cell proliferation and increased apoptosis were observed in WA-treated tumour-bearing mice in comparison with tumours of the control mice group found in Stan et al. [22]. The study by Szarcvelszic et al. (2014) [23] has found that WA can overcome the therapy resistance by targeting multiple cancer characteristics like cell death resistance, replicative immortality, invasion & metastasis and also cell proliferation. In the study of Imamura M et al. (2018) [24], they say that CEA is useful for predicting the progress of breast cancer patients. So, in our study, we have used CEA levels to analyze the prognosis of breast cancer in Wistar rats treated with Propolis & WA in combination to see the maximum effect of anticancer activity.

## Conclusions:

Data shows that the combination studies of propolis & WA in tumour-bearing female rats have shown an excellent progression compared to the female rats, which is not bearing breast cancer. Further study is required to use this combination in human trials/treatment.
